# Puzzling With Online Games (BAM-COG): Reliability, Validity, and Feasibility of an Online Self-Monitor for Cognitive Performance in Aging Adults

**DOI:** 10.2196/jmir.2860

**Published:** 2013-12-03

**Authors:** Teun Aalbers, Maria A E Baars, Marcel G M Olde Rikkert, Roy P C Kessels

**Affiliations:** ^1^Radboud University Medical CenterDepartment of Geriatric MedicineNijmegenNetherlands; ^2^Radboud University Medical CenterRadboud Alzheimer CenterNijmegenNetherlands; ^3^Radboud University NijmegenDonders Institute for Brain, Cognition and BehaviourNijmegenNetherlands; ^4^Radboud University Medical CenterDepartment of Medical PsychologyNijmegenNetherlands

**Keywords:** cognitive testing, brain aging, games, validity, reliability, self-monitoring, Internet, eHealth

## Abstract

**Background:**

Online interventions are aiming increasingly at cognitive outcome measures but so far no easy and fast self-monitors for cognition have been validated or proven reliable and feasible.

**Objective:**

This study examines a new instrument called the Brain Aging Monitor–Cognitive Assessment Battery (BAM-COG) for its alternate forms reliability, face and content validity, and convergent and divergent validity. Also, reference values are provided.

**Methods:**

The BAM-COG consists of four easily accessible, short, yet challenging puzzle games that have been developed to measure working memory (“Conveyer Belt”), visuospatial short-term memory (“Sunshine”), episodic recognition memory (“Viewpoint”), and planning (“Papyrinth”). A total of 641 participants were recruited for this study. Of these, 397 adults, 40 years and older (mean 54.9, SD 9.6), were eligible for analysis. Study participants played all games three times with 14 days in between sets. Face and content validity were based on expert opinion. Alternate forms reliability (AFR) was measured by comparing scores on different versions of the BAM-COG and expressed with an intraclass correlation (ICC: two-way mixed; consistency at 95%). Convergent validity (CV) was provided by comparing BAM-COG scores to gold-standard paper-and-pencil and computer-assisted cognitive assessment. Divergent validity (DV) was measured by comparing BAM-COG scores to the National Adult Reading Test IQ (NART-IQ) estimate. Both CV and DV are expressed as Spearman rho correlation coefficients.

**Results:**

Three out of four games showed adequate results on AFR, CV, and DV measures. The games Conveyer Belt, Sunshine, and Papyrinth have AFR ICCs of .420, .426, and .645 respectively. Also, these games had good to very good CV correlations: rho=.577 (*P*=.001), rho=.669 (*P*<.001), and rho=.400 (*P*=.04), respectively. Last, as expected, DV correlations were low: rho=−.029 (*P*=.44), rho=−.029 (*P*=.45), and rho=−.134 (*P*=.28) respectively. The game Viewpoint provided less desirable results with an AFR ICC of .167, CV rho=.202 (*P*=.15), and DV rho=−.162 (*P*=.21).

**Conclusions:**

This study provides evidence for the use of the BAM-COG test battery as a feasible, reliable, and valid tool to monitor cognitive performance in healthy adults in an online setting. Three out of four games have good psychometric characteristics to measure working memory, visuospatial short-term memory, and planning capacity.

## Introduction

With the rise of the Internet and the introduction of eHealth, the new research area of online health care has evolved rapidly over the last decade [1]. The field of research focusing on public health promotion is no exception [2]. Also, and already for a slightly longer period of time, the gaming industry has established itself as a major global industry [3]. Nowadays, eHealth and “serious gaming” are increasingly intertwined and more researchers are venturing into the realm of (online) game research. In turn, game developers show heightened interest in supporting and helping solve scientific research and societal issues [4]. For example, games are used to assist in stroke rehabilitation [5], in programs aimed at the prevention of youth obesity [6], and in enhancing gait balance in nursing home residents [7].

From a health-behavior change perspective, both eHealth and gaming are of high interest. Widespread Internet access provides the behavior-change researcher with the platform necessary to reach large populations. In Europe and North America, Internet penetration ranges between 63.2-78.6% of the total population [8]. With its massive reach, online gaming has long since shifted from being a typical pastime for younger generations to serving millions of gamers of every age, race, sex, and cultural background [9].

An important drawback of the Internet is that its content has to be fast and entertaining [10,11]. When researchers consider using the Internet as their medium and want to profit from its enormous reach, their interventions and evaluation methods should comply with these characteristics. Therefore, there is a need for quick, easily accessible, and attractive applications and instruments that provide the user with direct feedback [12]. If an intervention fails to do so, it will be difficult to recruit a sufficient number of participants. Also, dropout rates may be high, which will subsequently heavily affect the power of a study [13] .

The effects of aging on cognitive functions have been studied increasingly [14,15]. Typically, this has been done by both paper-and-pencil and offline computer-assisted neuropsychological testing [16]. One of the domains within the area of eHealth involves online assessment and monitoring of cognitive (dys)function [17]. Quantifying cognitive performance in tangible measures that are readily interpretable for neuropsychologists and patients alike has gained increasing interest and cognitive training programs like Lumosity have experienced a steep rise in popularity [18]. Now that intervention studies are scaling up in the number of recruited participants, a demand exists for short and easy-to-use validated neuropsychological tests [19]. Traditional person-to-person neuropsychological testing may in this respect often be inefficient from a time and cost perspective [20,21] and certainly does not meet the criteria for successful use in an online environment.

Online cognitive testing has already been proven valid and reliable in children aged 10-12 years [20], as well as adult and older populations ranging from 18-80 years of age [17,22]. We set out to develop an online self-monitor for cognitive functioning in people aged 40 years and older—the BAM-COG (Brain Aging Monitor-Cognitive Assessment Battery). The BAM-COG consists of four easily accessible, short yet challenging puzzle games that can be completed online, aimed to assess key aspects of cognitive function that are susceptible to aging-related changes, that is, working memory, executive function, and episodic memory. This empirical validation study consisted of two parts. First, we examined the alternate forms reliability and, second, we studied convergent and divergent validity of the BAM-COG. Also, reference values are presented from a sample of 397 adults aged 40-85 years.

To our knowledge, this is the first study to describe, validate, and examine an online self-monitor for cognitive functioning that makes use of visually attractive, easy-to-instruct puzzle games. The BAM-COG was not developed as a diagnostic tool (eg, for the assessment of pathological cognitive aging such as dementia), nor was it designed to predict cognitive decline over time. The aim of the BAM-COG was to enable users to establish their cognitive performance and to monitor their personal cognitive development over time. This is of major importance because it greatly increases the possibilities of online research on cognitive functioning, it increases reach, and it decreases costs both monetary and in time.

The hypotheses for this study are that the BAM-COG games have good alternate forms reliability and that the face and content validity of the four newly developed puzzle games of the BAM-COG transfer into good convergent and divergent validity, compared with standard paper-and-pencil and computer-assisted cognitive assessment.

## Methods

### Population

We set out to validate the BAM-COG in a cohort of community-dwelling individuals aged 40 years and older**.** Rationale for the 40-year cut-off point is that from approximately this age onwards normal cognitive aging is firmly evidenced [23]. The only inclusion criterion, apart from age, was that participants had adequate Internet access. Within the given age restrictions, the target population was unrestricted since we searched for a study population representative of the general population. No regional, ethnic background, sex, or language restrictions were applied, although the website description was only available in Dutch. Participants for Part 1 of the study were recruited online through several websites, social media, and blogs. A convenience sample was recruited for Part 2 of the study using flyers in community centers, shopping areas, mid-sized regional organizations, and senior centers. Furthermore, the study received national radio and newspaper attention, which resulted in the recruitment of participants as well.

### Study Design

The research website was available to participants for four months. Upon enrollment, we registered sex, age, and education level—the latter ranging from 1-8, where 1 is the lowest value (elementary school) and 8 is the highest value (university level education; see [22] for the Dutch system which is similar to the ISCED [International Standard Classification of Education] standards from the United Nations [24]). The online games could be completed in the uncontrolled setting of the participants’ day-to-day lives [21]. Once participants were logged in, they played the BAM-COG games for the first time. An automated reminder system prompted the participant to visit the website again after 14 and 28 days to perform the second and third round of BAM-COG games.

On their first two visits, participants performed the same BAM-COG games (see Table 1 for more information on the BAM-COG games). In the third round, they performed a different batch of BAM-COG games, thus playing different trials with approximately the same difficulty. To check whether the different batches did not differ with respect to difficulty, we performed alternate forms reliability (AFR) analyses (see Statistical Analyses). In total, there were three different batches of trials. A participant was randomly assigned to any of the six possible sequence groups (1-1-2, 1-1-3, 2-2-1, 2-2-3, 3-3-1, or 3-3-2) by an online random placement script. After completing all three rounds, a participant was awarded a promotional code with a value of €4.99 (US$6.75) that could be used for a one-month subscription to a puzzle website.

There were two parts in this study. Part 1 involved the data collection for AFR analyses and reference values, which was done exclusively via the Internet. Participants in Part 1 were estimated to need approximately 45 minutes per session to complete the BAM-COG. In total, after three rounds of BAM-COG puzzles within 28 days, participants were estimated to have spent approximately 135 minutes on the BAM-COG. This group will be abbreviated as “Online group” from this point on. Part 2 involved the data collection necessary to calculate the BAM-COG’s convergent (CV) and divergent validity (DV). For this procedure, in addition to playing the BAM-COG games online, participants visited the Radboud University Medical Center (RUMC) once (this group will be abbreviated as the “RUMC group”). This group of participants performed both computerized cognitive tests (subtests from the Cambridge Automated Neuropsychological Test Battery or CANTAB) and paper-and-pencil neuropsychological tests (PnP) (see Table 2 for an overview of the tests and Multimedia Appendix 1 for a more detailed description of the BAM-COG). Specific subtests were related to the individual BAM-COG’s cognitive constructs by consultation with experienced neuropsychologists (MAEB, RPCK; see Table 2 for overview of used measures of comparison). Order of the offline testing (CANTAB first vs PnP tasks first) was randomized by flipping a coin. BAM-COG results from participants in Part 2 are also included in the results of Part 1. Duration of the test session was approximately 90 minutes per participant. In addition to the 135 minutes spent on the BAM-COG measurements, participants in Part 2 were estimated to have spent about 225 minutes on the BAM-COG validation study.

For the group of participants visiting the RUMC, two additional inclusion and exclusion criteria were applied. Potential participants were excluded if they had a score ≤24 on the Mini-Mental State Examination (MMSE [25]) to make sure none of the participants had any symptoms of neurodegenerative disease [16]. To ensure that participants were capable of working with the CANTAB touch screen and test environment, the session started with performing the CANTAB Motor Screening Task where participants need to touch a flashing “x” stimulus on the screen as quickly and accurately as possible. If participants failed to either comprehend or execute this task, they were excluded from further participation. Since this study design was, in part, focused on gathering reference values, current participants did not receive feedback on their individual scores in comparison to their peers. After completing the three measurements, participants did not have continued access to the games, because the BAM-COG was not designed to be a training instrument, but an assessment instrument. This resembles the manner in which it primarily should be used in further practice.

**Table 1:** BAM-COG (Brain Aging Monitor–Cognitive Assessment Battery) game details.

BAM-COG game	Cognitive domain	Total levels^a^	Range of scores	Short description
Conveyer Belt	Working memory	7	4-10	This game shows a participant a grocery list on screen. After 1 second, the conveyer belt turns on. Groceries run down the belt and participants need to select only those products that are on their list.
Sunshine	Visuospatial short-term memory	8	3-10	In this game, a sun creates visual patterns in a 5x5 cloud matrix. This visual pattern dissolves and, after it has completely disappeared, participants are asked to reproduce this pattern in the exact same order as it initially appeared on screen.
Viewpoint	Episodic recognition memory	8	1-8	This game presents a 5x5 matrix filled with stimuli (asterisks) to the participant. The participant gets 3 seconds to memorize this presented pattern before it disappears from the screen. After 3 seconds, 3 answer possibilities appear on screen from which the participant is to pick the answer that is an exact match to the previously shown matrix.
Papyrinth	Executive function - planning	5	3-7	This game starts with presenting the participant with a scrambled path. The participants task is to unscramble the path so their pawn can move from start to finish unobstructed. Clearing the route is done by sliding columns and rows in the correct order so that all pieces of road end up connected to each other.

^a^Excluding the practice level.

**Table 2:** BAM-COG^a^ domains and proposed matching computerized and paper-and-pencil cognitive tests.

BAM-COG game (domain)	CANTAB^b^	Paper and pencil
Conveyer Belt (working memory)	Spatial Working Memory [26]	Letter-Number Sequencing Task from WAIS-III^c^ [27]
Sunshine (visuospatial short-term memory)	Spatial Span [26]	Spatial Span subtest from WMS-III^d^ [28]
Viewpoint (episodic recognition memory)	Pattern Recognition [26]	Continuous Visual Memory Task [16,29-31]
Papyrinth (planning)	Stockings of Cambridge [26]	Zoo Map Task, part of the BADS^e^ [16,32]

^a^BAM-COG: Brain Aging Monitor–Cognitive Assessment Battery. For a short description of the BAM-COG games, see Multimedia Appendix 1.

^b^CANTAB: Cambridge Automated Neuropsychological Test Battery.

^c^WAIS-III: Wechsler Adult Intelligence Scale, third edition.

^d^WMS-III: Wechsler Memory Scale, third edition.

^e^BADS: Behavioral Assessment of the Dysexecutive Syndrome.

### Sample Size Calculation

According to our sample size calculations for CV and DV, we needed 37 participants for Part 2 (alpha error probability <.05, power (1-beta error probability =.8) of our study. Sample size calculation was performed using GPower 3.1 [33].

### Instruments

The BAM-COG consists of four puzzle games developed to measure working memory, visuospatial short-term memory, episodic recognition memory, and executive function-planning (see Table 1 for game details). Every game started with brief and clear instructions as to what the participant should expect. In an attempt to maximize comprehension of the instructions, the written instructions were accompanied by actual game screenshots. After the mandatory instructions, participants performed one practice trial to further familiarize themselves with the game. Following this first practice trial, the actual test commenced. Each level of each game consisted of three trials. To advance to the next level, at least two out of three trials had to be completed successfully. If a participant failed to successfully complete two or three trials, a “game over” screen appeared and the participant was linked back to the main screen where the next game could be selected. For an overview of the games and their instructions, see Multimedia Appendix 1. Multimedia Appendices 2-5 include short videos of the BAM-COG game play. Scores for the Conveyer Belt, Sunshine, and Papyrinth games were the total number of stimuli or moves that needed to be processed. For the Viewpoint game, the score was the number of levels successfully completed.

### Measures of Comparison

Subjects in the RUMC group also participated in tasks from the CANTAB and PnP tasks matched for the BAM-COGs cognitive domains (see Table 2). All these games were carefully selected to mimic the cognitive domains primarily relied on in the BAM-COG games as closely as possible.

### Instrument Development

Based on expert opinion from two neuropsychologists, a geriatrician, a public health researcher, and a professional game-design team, the four puzzle games were considered to cover the chosen cognitive constructs of working memory, visuospatial short-term memory, episodic recognition memory, and planning. After this initial assessment, the instrument outline was discussed with a broader group of health care professionals consisting of neuropsychologists, epidemiologists, public health care researchers, and general psychologists. It was agreed that from a content point of view, it would be impossible to cover every cognitive domain that decreases in functionality across the lifespan, when fast and easy access are key criteria. It was decided that choosing three executive functions and one specific memory function, all of which have been established to decline in normal aging and neurodegenerative syndromes [23,34-37], would provide good insight into overall aging patterns.

### Statistical Analysis

Alternate forms reliability (AFR) was determined to compare the three batches of BAM-COG games, administered at different time points. Every batch resembles a parallel version of the BAM-COG containing an equal number of levels and trials. Theoretically, these batches do not differ from one another in difficulty. The AFR was determined with an intraclass correlation (ICC: two-way mixed; consistency at 95%) on the results of the second and third round performances of the participants. With respect to interpretation of the ICCs, we needed to take into consideration that the study was executed outside of a clinical laboratory setting where people could be easily distracted, which may affect the test’s reliability. Therefore, ICC values between .4 and .6 were considered sufficient to support AFR for the BAM-COG. This is in line with another online validation study [17]. Also, note that no specific cut-off scores for ICCs exist [38].

To further analyze possible systematic differences between measurements, Bland-Altman plots were calculated. In these plots, the differences between two sessions were plotted against their mean. Furthermore, the scores’ means and limits of agreement were calculated as the mean of the difference between the two measurements ±2 SD of these differences. The standard error of measurement and the 95% confidence intervals for the mean difference between the two measurements were also calculated. If the 95% confidence interval does not include zero, this indicates a systematic and undesirable change in the mean [39].

The CV determines whether the cognitive domain supposedly measured by the BAM-COG game is actually assessed, using validated cognitive tasks as gold standards. In contrast, the DV examines to what extent the BAM-COG correlates with cognitive domains it should not correlate with. By comparing the BAM-COG game scores to a non-related cognitive construct (in this study, IQ scores derived from the Dutch version of the National Adult Reading Test, NART), the distinctive capacities of the BAM-COG are established. Due to non-normal data distribution on BAM-COG outcome measures and small samples, both CV and DV of the BAM-COG are calculated using a one-tailed Spearman’s rho correlation coefficient.

For interpretation purposes, the data from the three batches were aggregated into one measure for the calculation of CV and DV. This enables us to judge the task as one entity instead of three separate batches. Single test statistics were generated based on participants’ average game scores (for more information on scoring, see Instruments). Reference values are provided for the games to provide some insight into the expected distribution of scores in a normal aging population of people aged 40 years and older. For every analysis, participants with a raw test score of 0 were excluded. This was done as these participants had either viewed the instructions but not started playing or played only one or two trials out of the necessary three to advance to the next level.

This study was deemed exempt from formal ethical evaluation by the local medical ethics committee (region Arnhem-Nijmegen, registration number: 2011/490). All statistical analyses were performed using IBM SPSS Statistics for Windows, Version 20.0. The Bland-Altman plots were performed with GraphPad Prism version 5.03 for Windows.

### Feasibility

BAM-COG’s feasibility was assessed based on the total number of registrations and dropouts, the percentage of participants who played and completed the first, second, and third rounds, and examination of the score distributions for floor and ceiling effects.

## Results

### Participants

Through our research website, 641 participants were enrolled in this study of whom 124 (19.3%) were excluded as they did not fulfill the age criterion. Immediately after registering, each participant was asked to perform the BAM-COG test battery for the first time. A total of 76.8% (397/517) participants in this group played at least one game and were therefore eligible for analyses; 78.6% (312/397) of these were women. The mean age was 54.9 (SD 9.6) years and the modus of education level was 6 (range 1-8).

We recruited 56 participants to participate in Part 2 of the study. Of these 56 participants, 41 were willing to register online, with a mean age of 60.8 (SD 8.2) years, of whom 58.5% (24/41) were female with a modus of educational level of 7 (range 1-8). All participants were native Dutch speakers. All were able to successfully complete the CANTAB Motor Screening Task. In total, 21 (51.2%) of the 41 participants completed the CANTAB tasks first as compared to 20 (48.8%) of the 41 participants completing the PnP tasks first.

In Table 3, scores for the MMSE, NART-IQ, and mean BAM-COG scores are presented. Data from the three batches were pooled to get an overall average score on all four games. The RUMC group was significantly older (*t*_395_=3.78, *P*<.001) and had a higher education level (χ^2^_7_=33.8, *P*<.001). This resulted in higher overall test scores (except for Viewpoint) even though these differences only reached statistical significance in Sunshine. Since there was such a large inequality in gender distribution in our sample, we controlled for systematic differences between men and women on the raw BAM-COG scores. Using a Fisher Exact test, we found no significant differences (ranging from *F*_13_=18.68, *P*=.07 to *F*_19_=21.82, *P*=.19).

**Table 3:** Mean (SD) for age, MMSE^a^, NART-IQ ^b^, and BAM-COG^c^ scores and mode (range) for education for RUMC^d^ and online group.

	Online group	RUMC group
Age, years, mean (SD)	54.9 (9.6)	60.8 (8.2)
Education, mode (range)	6 (1-8)	7 (1-8)
MMSE, mean (SD)	--	29.4 (1.07)
NART-IQ, mean (SD)	--	123.2 (12.83)
Conveyer Belt score	5.95 (n=217)	6.33 (n=26)
Sunshine score	4.60 (n=236)	5.10 (n=24)
Viewpoint score	3.97 (n=306)	3.90 (n=28)
Papyrinth score	4.64 (n=152)	5.30 (n=21)

^a^MMSE: Mini Mental State Examination.

^b^NART-IQ: National Adult Reading Test–Intelligence Quotient.

^c^BAM-COG: Brain Aging Monitor–Cognitive Assessment Battery.

^d^RUMC: Radboud University Medical Center.

### Alternate Forms Reliability

Table 4 shows the AFR with their respective 95% confidence intervals for all four BAM-COG games. With the exception of Viewpoint, all games have good (>.4) to very good (>.6) AFR. To further clarify this relationship, Multimedia Appendix 6 shows the generated Bland-Altman plots. These also show that, with the exception of the Viewpoint game, the error bias does not deviate far from zero. This ascertains the absence of systematic error between the second and third round measurements.

**Table 4:** Alternate forms reliability (AFR) of BAM-COG^a^ games in intraclass correlations (ICC^b^).

BAM-COG game	AFR	95% CI
Conveyer Belt (n=55)	.420	0.17-0.62
Sunshine (n=78)	.426	0.23-0.59
Viewpoint (n=101)	.167	−0.04 to 0.36
Papyrinth (n=37)	.645	0.41-0.80

^a^BAM-COG: Brain Aging Monitor–Cognitive Assessment Battery.

^b^All ICC values >.4 are considered to support sufficient AFR.

### Convergent and Divergent Validity

With the exception of Viewpoint, the BAM-COG games have good (>.4) to very good (>.6) CV in comparison to both the CANTAB and PnP tasks (see Table 5). Conversely, as hypothesized, all games also show good (<.2) DV with an unrelated overall measure of IQ. Please note that a poor AFR for Viewpoint also translates into poor CV and DV values.

To control whether the individual games did not heavily load on the same cognitive domain, we performed Spearman correlation analysis using aggregated game scores. As was expected with a large sample, most correlations are significant. However, the size of the correlations range from very small (rho=.143, *P*=.056), between Conveyer Belt and Viewpoint, up to medium small (rho=.406, *P*<.001), between Sunshine and Papyrinth.

**Table 5:** Convergent and divergent validity of BAM-COG^a^ games (Spearman rho’s correlation coefficient).

BAM-COG game	Convergent validity^b^		Divergent validity^c^		
	Cognitive test	rho (*P* value)	Cognitive test		rho (*P* value)
**Conveyer Belt (n=26)**					
	WAIS-III^d^ Letter Number Sequencing	.577 (.001)		National Adult Reading Test	−.029 (.44)
	Spatial Working Memory	−.577 (.001)			
**Sunshine (n=24)**					
	WMS-III^e^ Spatial Span Task	.669 (<.001)		National Adult Reading Test	−.029 (.45)
	Spatial Span	.620 (.001)			
**Viewpoint (n=28)**					
	Continuous Visual Memory Test	.202 (.152)		National Adult Reading Test	−.162 (.21)
	Pattern Recognition	−.157 (.212)			
**Papyrinth (n=21)**					
	BADS^f^ Zoo Map	.400 (.036)		National Adult Reading Test	−.134 (.28)
	Stockings of Cambridge	.424 (.028)			

^a^BAM-COG: Brain Aging Monitor–Cognitive Assessment Battery.

^b^All convergent validity values of rho≥.4 are considered to support good CV; values of rho≥.6 are considered very good.

^c^All divergent validity values of rho<.2 are considered to support good DV.

^d^WAIS-III: Wechsler Adult Intelligence Scale, third edition.

^e^WMS-III: Wechsler Memory Scale, third edition.

^f^BADS: Behavioral Assessment of the Dysexecutive Syndrome.

### Reference Values

We present reference values for all games (Table 6) displaying the total number of times any given score was reached in all three batches.

**Table 6:** BAM-COG^a^ reference values.

	Conveyer Belt (n=217)		Sunshine (n=236)		Viewpoint (n=306)		Papyrinth (n=152)	
Score	Frequency	Percentage	Frequency	Percentage	Frequency	Percentage	Frequency	Percentage
1	NA^b^	NA	NA	NA	145	27.5	NA	NA
2	NA	NA	NA	NA	57	10.8	NA	NA
3	NA	NA	75	19.7	32	6.1	57	25.3
4	78	24.4	148	38.9	90	17.1	82	36.4
5	100	31.3	79	20.8	70	13.3	27	12.0
6	26	8.1	55	14.5	41	7.8	15	6.7
7	43	13.5	15	3.9	13	2.4	44	19.6
8	58	18.2	6	1.7	79	15	NA	NA
9	12	3.8	2	0.5	NA	NA	NA	NA
10	2	0.7	0	0	NA	NA	NA	NA

^a^BAM-COG: Brain Aging Monitor–Cognitive Assessment Battery.

^b^NA: Not Applicable, as this score is not a possible outcome for this game.

### Feasibility

The number of registrations totaled 641 participants. The BAM-COG received nationwide attention on two national radio shows and in several regional and national newspapers and magazines. Of the 517 eligible participants, only 397 participants played at least one game out of any of the three batches (76.8%).

The Conveyer Belt game was played most at all three assessments (314, 143, and 107 times respectively) and Papyrinth was played the least frequently (189, 123, and 87 times respectively). On average, 75.7% of participants played all four games and, from the participants that finished the last game on a previous round, on average 80.7% returned to play the next round.

Only 8 participants quit while in the middle of playing a game. All the other participants continued until the “game over” message appeared and either continued with the next game or decided to quit playing after this message. The 8 participants who dropped out all stopped while playing Papyrinth, which is the only game that does not have an integrated time limit.

No real floor or ceiling effects were present in the data. The only possible exception to this may be a slight ceiling effect on Papyrinth and Viewpoint (with 19.6%, 44/225 and 15.0%, 79/527 respectively, completing the highest level). Otherwise, the percentages of participants completing the tasks were very low (0.5%, 2/380 and 0.7%, 2/319 respectively).

## Discussion

### Principal Findings

This article provides substantial support for the use of the BAM-COG game battery as an online self-monitor for cognitive performance. Three out of four games appear to be adequate measures of the related cognitive concepts (working memory, visuospatial short-term memory, and planning). Conveyer Belt, Sunshine, and Papyrinth all have good alternate forms reliability and turned out to be feasible for use in aging adults. Furthermore, they all have good to very good convergent and divergent validity and reference values for the games are now available. Since all games were designed to measure some form of cognitive domains, it stands to reason that their correlations are statistically significant. Their size, however, is either considerably smaller or equal to the task correlations with outside gold-standard measurement tools. The game Viewpoint, designed to assess episodic recognition memory, did not have an adequate validity and reliability and is not suitable for inclusion in an online assessment battery. In addition, a strength of our setup are the correlations of the BAM-COG scores with the gold-standard CANTAB and PnP tasks. The fact that the BAM-COG games proved to be solid measures of the intended cognitive domains provides good hope that replication of these results is possible in other samples and the BAM-COG can be put to use for its intended purpose.

### Limitations

Even though the current findings are promising with respect to the BAM-COG’s applicability, some adjustments can be recommended on the basis of these results. First, we occasionally received feedback of technical difficulties, in particular with the performance of the Conveyer Belt game. Small-sized stimuli (in this case, groceries such as apples and pears) appeared difficult to click resulting in unintentional missed responses. However, although we cannot fully rule out technical issues on some remote systems, this may have also been due to suboptimal mouse handling by individual participants. This explanation is likely since neither the software developers nor the researchers have been able to replicate this problem on different systems with different operating systems and Internet browsers. Moreover, the problem did not emerge so frequently (n=19 out of n=314) that it would have severely influenced the outcomes of our analyses. Second, feedback was given that there is a need for additional practice levels. Apparently just one trial to get acquainted with the task was not always enough for all participants to fully comprehend what was requested of them. This may have resulted in a slight underachievement in average scores. In a future release of the BAM-COG battery, this can easily be taken into account. Third, regardless of our follow-up efforts (one additional phone call and one personal reminder email), 15 participants in the RUMC group failed to register online even after they had visited the memory clinic. Reasons for this dropout could have been a sole interest in the neuropsychological screening at the research center, time restrictions, loss of motivation, or the relative ease with which reminder emails and online interventions can be ignored and forgotten. Additionally, the limited amount of personal contact with the researchers and the ease of the registration process may increase attrition [40,41], as well as technical or computer-access problems, physical illness, burden of the program, the static structure, and low adaptation to user preferences [42,43]. This again stresses that high dropout rates are an important issue to consider when setting up Internet-based studies. However, since the characteristics of the group of dropouts did not differ in any way from the other registered participants, we do not feel this has significantly affected the current results.

In the interpretation of these results, we need to take the naturalistic setting in which the games were performed into account. That is, laboratory studies in which results are produced under highly controlled conditions typically result in higher ICCs and correlations. The BAM-COG assessments in this study have all been performed in the participants’ home environment without any supervision by the research team. Because the BAM-COG is not designed to be used in a laboratory setting, we feel the present design is a valid approach to examine its feasibility, validity, and reliability. If biased, the performance presented in this study may be an underestimation of the real reliability and validity of the BAM-COGs tasks [38]. Therefore, we feel we can validly conclude that the BAM-COG is an adequate online self-monitor for cognitive performance.

The fact that our population consisted mainly of women (78.6%, 312/397 and 58.5%, 24/41 for Part 1 and Part 2 respectively) somewhat decreases the external validity of this study. However, this type of research and these types of puzzle games have previously been shown to attract more female participants than males [9,17,22]. Also, the notion that not all participants finished (all) the games has consequences for the way ceiling and floor effect results should be interpreted. It remains possible that the participants not starting or dropping out in level 1 are, in fact, experiencing a floor effect. Finally, it should be mentioned that the RUMC group differed from the online group, as the RUMC group was both older and better educated. This resulted in slightly higher average test scores. Further research in a more balanced sample could strengthen the conclusions drawn and external validity for the BAM-COG battery and validation studies with other cognitive measures should be performed to replicate the present results.

### Conclusions

In sum, this study provides evidence for the use of the BAM-COG test battery as a feasible, reliable, and valid tool to monitor cognitive performance in healthy adults in an online setting. Three out of four games were found to have good to very good psychometric characteristics to measure working memory, visuospatial short-term memory, and planning capacity. It should be stressed that the results can by no means be used to either diagnose neurodegenerative disorders or predict cognitive performance. The BAM-COG is suitable for use in practice for online monitoring cognition and stimulating eHealth interventions for healthy brain aging.

## Multimedia Appendix 1

Overview of the BAM-COG games.

## Multimedia Appendix 2

Short video of BAM-COG’s game play - Conveyer Belt.

## Multimedia Appendix 3

Short video of BAM-COG’s game play - Sunshine.

## Multimedia Appendix 4

Short video of BAM-COG’s game play - Viewpoint.

## Multimedia Appendix 5

Short video of BAM-COG’s game play - Papyrinth.

## Multimedia Appendix 6

Overview of the Bland-Altman plots for alternate forms reliability.

## Abbreviations

AFR: alternate forms reliability
BAM-COG: Brain Aging Monitor – Cognitive Assessment Battery
CANTAB: Cambridge Automated Neuropsychological Test Battery
CV: convergent validity
DV: divergent validity
ICC: intraclass correlation
IQ: Intelligence Quotient
ISCED: International Standard Classification of Education
MMSE: Mini Mental State Examination
NART: National Adult Reading Test
RUMC: Radboud University Medical Center
