# Supplementary material for: Puzzling With Online Games (BAM-COG): Reliability, Validity, and Feasibility of an Online Self-Monitor for Cognitive Performance in Aging Adults
Source: J Med Internet Res. 2013 Dec 3;15(12):e270. doi: 10.2196/jmir.2860 (PMC3868977; doi:10.2196/jmir.2860)
Supplement: Supplementary file 1 [file jmir_v15i12e270_app1.pdf]

The four puzzles used for the BAM-COG are online at www.spellenonderzoek.nl (website in Dutch). The description in this appendix should provide the reader with a fair overview of what the puzzles contain.

Conveyer Belt

Conveyer belt, a puzzle game relying mostly on working memory, shows a participant a grocery list on screen. After one second the conveyor belt turns on. Groceries run down the belt and participants need to select only those products that are on their list.

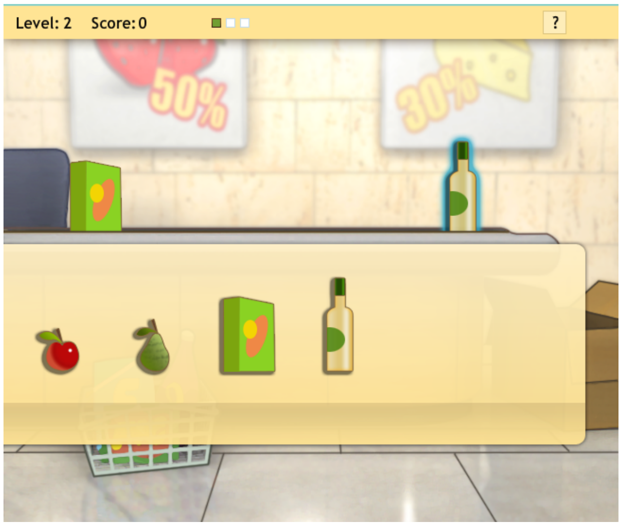

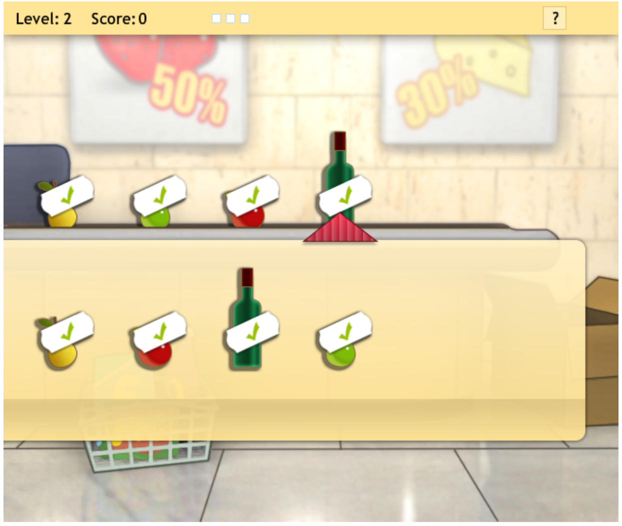

Conveyer Belt Instructions:

1. In the game ‘conveyer belt’ you get to see a list of groceries. You have to select the groceries on this list when they come down the conveyer belt.
2. You can select a product by clicking it on the conveyer belt. Make sure to click them before they drop into the box at the end of the belt!
3. Make sure that you do not select too many groceries or that you don’t select all the groceries
4. We start out with a short practice round with only three types of groceries.
5. When you don’t succeed in successfully completing two out of three trials you will be game over.

Sunshine

Sunshine, a puzzle game relying mostly on visuospatial short-term memory, creates visual patterns in a 5x5 matrix. This visual pattern dissolves gradually and after it is completely gone from the screen participants are asked to reproduce this pattern in the exact same order as it initially appeared on screen.

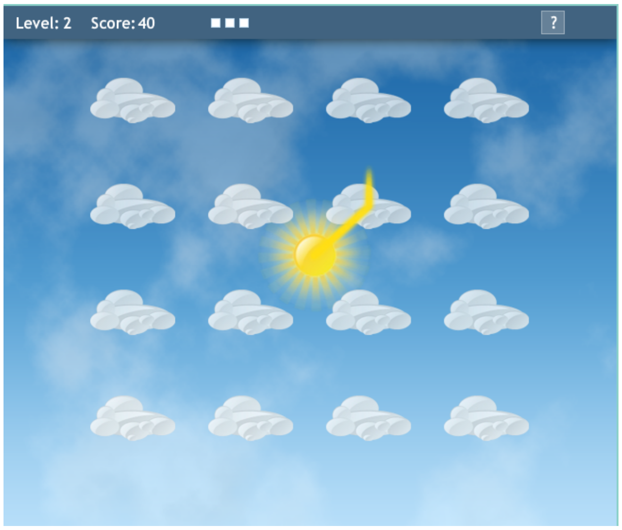

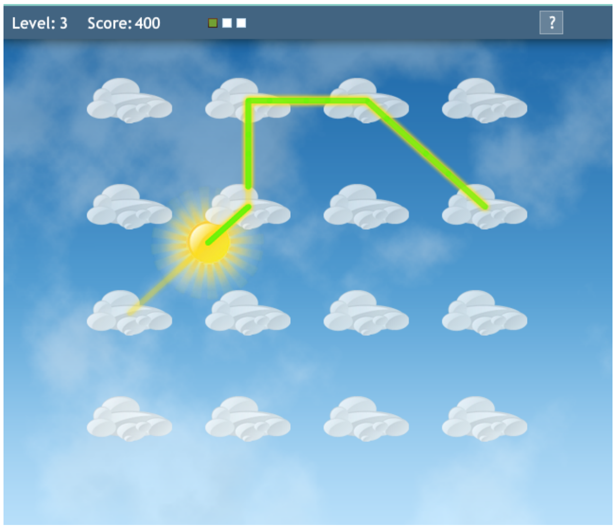

Sunshine Instructions:

1. The game ‘sunshine’ starts with a sun drawing a line between several clouds in a 4x4 grid. It is up to you to memorize the pattern of the line drawn.
2. It is your task to draw the exact same line as the sun did as soon as the sun disappears from the screen. You can do this by clicking the clouds in the correct order.
3. Please note: make sure that when you start your clicking sequence that the first cloud is actually selected (it will light up slightly).
4. You cannot take back steps. The trial end as soon as you have drawn an equal number of line pieces as drawn by the sun. We start out with a short practice round with a short line of only three line pieces.
5. When you don’t succeed in successfully completing two out of three trials you will be game over.

Viewpoint

Viewpoint, a puzzle game relying mostly on episodic recognition memory, presents a 5x5 matrix filled with stimuli to the participant. The participant gets three seconds to memorize this presented pattern before it disappears from the screen. After three seconds three possible answers appear on screen from which the participant is to pick the answer that is an exact match to the previously shown matrix.

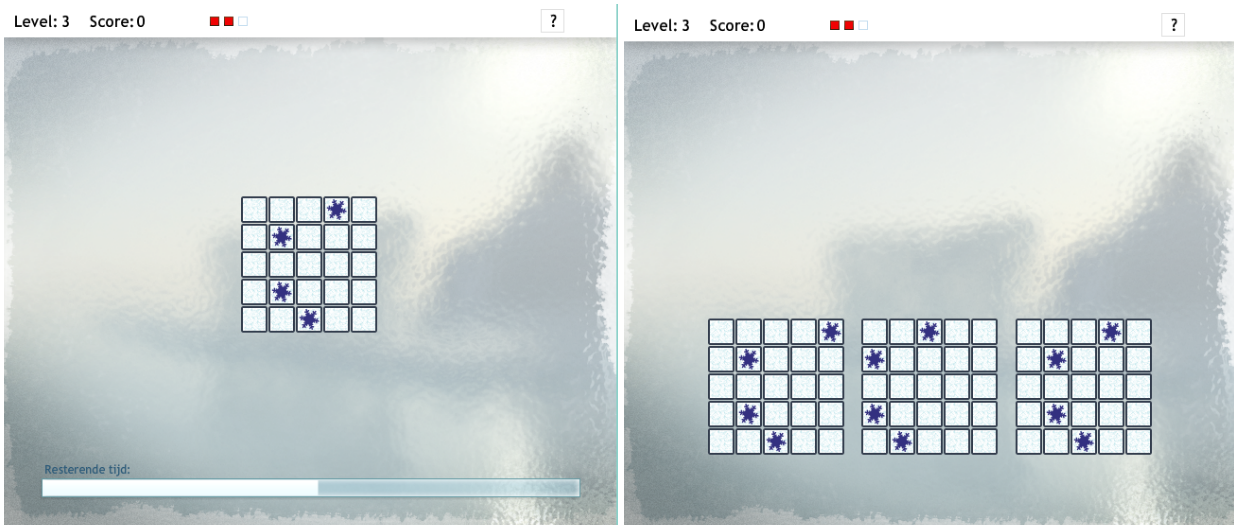

Viewpoint Instructions:

1. In the game ‘viewpoint’ you get to see a 5x5 matrix that contains a predetermined pattern made out of asterisks. You need to memorize this pattern.
2. After three seconds the pattern disappears from the screen and the screen goes blank.
3. The blank screen is replaced with three answer possibilities. Only one of these three patterns is an exact copy of the originally shown pattern. You’re supposed to select the exact copy.
4. We start out with a short practice round with only three groceries.
5. When you don’t succeed in successfully completing two out of three trials you will be game over.

Papyrinth

Papyrinth, a puzzle game relying mostly on planning, starts with presenting a scrambled route to the participant. The participants task is to complete the route so their pawn can move from start to finish unobstructed. Clearing the route is done by sliding the columns and rows in such an order that all pieces of road end up connected to each other.

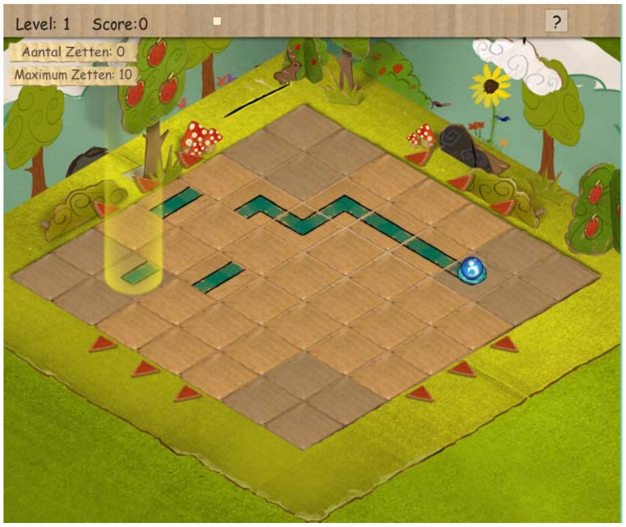

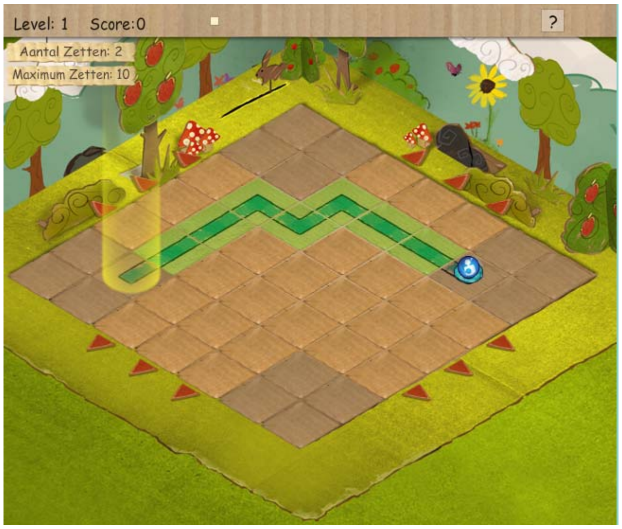

Papyrinth Instructions:

1. In Papyrinth you have to complete a route that is scrambled.
2. The goal of Papyrinth is to complete the route so that there is a unobstructed path from the start (the logo) to the finish (the yellow pillar).
3. You can complete the path by clicking the red arrows on the side of the rows and columns. Try to complete the pathway in as little moves as possible.
4. You can only move the entire row or column at once. Plan the moves you need to make strategically before actually clicking the arrows.
5. For every level Papyrinth tells you the maximal amount of moves you are allowed to make before you should have solved the puzzle.
6. When you do not succeed to successfully complete two out of three trials on any given difficulty the game is over.
