# Supplementary figures and images for: Puzzling With Online Games (BAM-COG): Reliability, Validity, and Feasibility of an Online Self-Monitor for Cognitive Performance in Aging Adults
Source: J Med Internet Res. 2013 Dec 3;15(12):e270. doi: 10.2196/jmir.2860 (PMC3868977; doi:10.2196/jmir.2860)

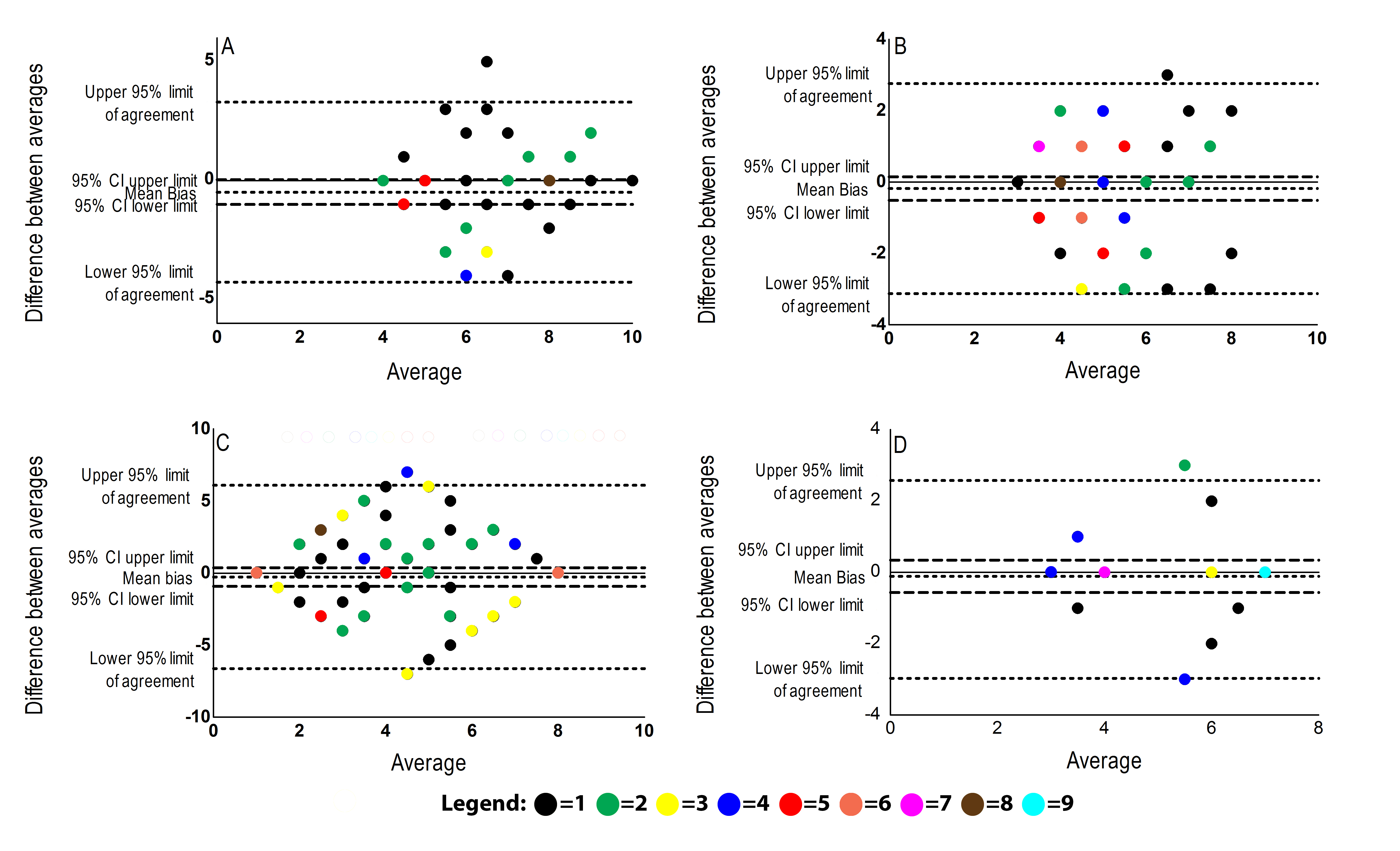

Supplement: Supplementary file 6 [file jmir_v15i12e270_app6.jpg]
